# I-GhostNetV3: A Lightweight Deep Learning Framework for Vision-Sensor-Based Rice Leaf Disease Detection in Smart Agriculture

**DOI:** 10.3390/s26031025

**Published:** 2026-02-04

**Authors:** Puyu Zhang, Rui Li, Yuxuan Liu, Guoxi Sun, Chenglin Wen

**Affiliations:** 1 College of Electronic Information Engineering, Guangdong University of Petrochemical Technology, Maoming 525000, China; zhangpy@gdupt.edu.cn (P.Z.); lirui@gdupt.edu.cn (R.L.); 2 Guangdong Provincial Key Laboratory of Petrochemical Equipment Fault Diagnosis, Guangdong University of Petrochemical Technology, Maoming 525000, China; liuyx@gdupt.edu.cn (Y.L.); wencl@gdupt.edu.cn (C.W.)

**Keywords:** rice disease classification, vision sensors, I-GhostNetV3, attention mechanisms, lightweight convolutional networks, smart agriculture

## Abstract

Accurate and timely diagnosis of rice leaf diseases is crucial for smart agriculture leveraging vision sensors. However, existing lightweight convolutional neural networks (CNNs) often struggle in complex field environments, where small lesions, cluttered backgrounds, and varying illumination complicate recognition. This paper presents I-GhostNetV3, an incrementally improved GhostNetV3-based network for RGB rice leaf disease recognition. I-GhostNetV3 introduces two modular enhancements with controlled overhead: (1) Adaptive Parallel Attention (APA), which integrates edge-guided spatial and channel cues and is *selectively inserted* to enhance lesion-related representations (at the cost of additional computation), and (2) Fusion Coordinate-Channel Attention (FCCA), a near-neutral SE replacement that enables efficient spatial–channel feature fusion to suppress background interference. Experiments on the Rice Leaf Bacterial and Fungal Disease (RLBF) dataset show that I-GhostNetV3 achieves 90.02% Top-1 accuracy with 1.831 million parameters and 248.694 million FLOPs, outperforming MobileNetV2 and EfficientNet-B0 under our experimental setup while remaining compact relative to the original GhostNetV3. In addition, evaluation on PlantVillage-Corn serves as a supplementary transfer sanity check; further validation on independent real-field target domains and on-device profiling will be explored in future work. These results indicate that I-GhostNetV3 is a promising efficient backbone for future edge deployment in precision agriculture.

## 1. Introduction

Rice is one of the most important food crops worldwide, and its yield and quality directly affect food security and the sustainable development of the agricultural economy [1]. Typical foliar diseases, such as rice blast, sheath blight, and bacterial leaf blight, impair photosynthetic capacity and accelerate premature plant senescence, leading to substantial yield and quality losses. Common yield reductions range from 10% to 30% and can be even higher during epidemic years [2,3]. In current production practice, field disease diagnosis primarily relies on visual inspection and manual grading by plant protection technicians or farmers. This approach is time-consuming, labor-intensive, and highly experience-dependent, suffering from strong subjectivity, poor consistency, and limited spatial coverage, which makes it difficult to meet the requirements of precise, large-scale disease control [4]. These challenges motivate automated, high-throughput recognition systems based on low-cost RGB cameras, smartphones, and UAV-mounted vision sensors, enabling scalable disease monitoring for smart agriculture.

The growing adoption of vision-based sensors, particularly RGB cameras and smartphones, has provided a cost-effective and scalable basis for real-time disease monitoring. However, compared with multispectral, thermal, or hyperspectral sensors that capture richer information, RGB devices record only three visible channels and are vulnerable to illumination and background variations [5]. This limitation is especially evident for early or small lesions whose color/texture contrast is weak and easily submerged by background clutter. Moreover, tropical climates (e.g., in Bangladesh for the RLBF dataset) can cause variations in leaf reflectance due to temperature, humidity, rainfall, and phenological stages [6,7]. Nonetheless, RGB remains the most accessible platform for smallholder farmers [8]. Therefore, the practical goal is not to replace RGB sensing but to develop lightweight and robust RGB-based models that can preserve lesion-relevant details under field-like disturbances and edge-device constraints.

Before deep learning, plant disease identification often relied on handcrafted cues (e.g., color, texture, and shape descriptors) combined with classifiers such as SVM or ANN [9,10]. Although such pipelines can work on small or controlled datasets, they tend to be sensitive to illumination changes, background complexity, and symptom variations, which limits their reliability in field scenarios. This has driven the adoption of end-to-end CNN models that learn task-specific representations directly from images and reduce reliance on manually designed features.

With the development of deep learning, particularly convolutional neural networks (CNNs), end-to-end feature learning has gradually become the mainstream approach for plant disease identification. CNNs can automatically learn multi-level semantic features from large-scale image datasets and generally outperform traditional methods based on handcrafted features, especially in lesion texture modeling, boundary capture, and background suppression. Building on this foundation, many studies have designed CNN-based frameworks for rice pest and disease detection, obtaining recognition accuracies exceeding 90% while keeping model structures relatively compact [11,12]. To address issues such as class imbalance and highly similar early symptoms across different diseases, channel and spatial attention mechanisms have been introduced to enhance the discrimination of fine-grained differences and easily confused categories.

In response to the challenges of lightweight design and feature enhancement for plant disease recognition on resource-constrained devices, various attention-based network improvements have been proposed. Cheng et al. introduced the ECA-ResNeXt model, embedding an Efficient Channel Attention (ECA) module into a ResNeXt backbone to significantly reduce parameters and computation while achieving 99.83% accuracy in rice disease recognition, thereby demonstrating the potential of lightweight architectures [13]. Sharma et al. proposed the AELGNet architecture, which integrates residual channel attention (RCA) and spatial attention (RSA) to collaboratively model local and global features, and achieved outstanding performance in medicinal plant leaf recognition [14]. In parallel, YOLOv5-based detection methods have been applied to real-time identification and localization of rice leaf diseases, improving adaptability and deployment efficiency in multi-lesion detection scenarios [15]. To balance recognition accuracy and computational efficiency, lightweight backbones such as MobileNet and EfficientNet have been widely adopted for disease recognition on mobile and embedded devices. For example, Elakya et al. [16] obtained 98.73% classification accuracy for rice diseases using an enhanced MobileNetV2 model while preserving a lightweight design, and Asvitha et al. [17] developed a smartphone-based rice pest and disease recognition system on MobileNetV3, achieving 93.75% accuracy and contributing to reduced crop losses. Despite these advances, most attention/fusion designs are general-purpose reweighting strategies; under field conditions with small, low-contrast lesions and heavy background clutter, a lightweight backbone may still under-represent boundary/edge evidence and amplify irrelevant background responses. This gap becomes more pronounced when compact networks rely on aggressive downsampling and inexpensive feature generation, where fine-grained spatial cues can be easily diluted.

First, many publicly available rice leaf disease datasets are collected in laboratory or laboratory-like environments with relatively uniform backgrounds and stable imaging conditions. Such datasets are limited in disease category diversity, sample scale, and field-related interference factors, which leads to considerable performance degradation when models trained on them are transferred to real production scenarios. In contrast, field images captured by RGB cameras or smartphones often contain small, sparsely distributed lesions with low contrast against complex backgrounds. Second, conventional CNN backbones inevitably lose high-resolution details and edge information during repeated downsampling, resulting in insufficient representation of small lesions and blurred lesion boundaries, which ultimately degrades recognition accuracy. Therefore, there is a need for lightweight models that can be effectively deployed on edge devices associated with vision sensors, while preserving fine-grained lesion details in complex field environments.

Motivated by these observations, we do not claim a novel architecture; instead, we target incremental yet practical improvements on a strong lightweight baseline (GhostNetV3) by introducing two plug-in attention modules with controlled overhead. The technical goal is to (i) reinforce boundary-sensitive lesion cues that are vulnerable in compact backbones, and (ii) strengthen spatial–channel interaction for background suppression while avoiding heavy multi-scale fusion. Importantly, the overall computation is controlled by using FCCA as a near-neutral SE replacement throughout the backbone and inserting APA only at a few stage outputs when boundary-sensitive cues are most needed.

This study aims to incrementally improve the accuracy and robustness of rice leaf disease identification in field settings while balancing model complexity and deployment cost for edge-oriented smart agriculture. To this end, we present I-GhostNetV3, a lightweight rice leaf disease classification model built upon GhostNetV3 and tailored to complex field scenarios with strict constraints on parameter size and computation.

Two pluggable attention modules, APA and FCCA, are designed to improve the accuracy–efficiency trade-off under a controlled compute budget. APA adopts a parallel design that combines an edge-guided spatial enhancement branch and an efficient channel recalibration branch, followed by gated fusion and residual refinement, to compensate for boundary/detail attenuation in compact feature maps; due to its non-negligible overhead, APA is selectively inserted at a few stage outputs when boundary-sensitive cues are most needed. FCCA couples direction-aware coordinate attention with efficient channel attention and performs lightweight channel-wise soft fusion, serving as a near-neutral drop-in replacement of SE to improve spatial–channel coupling and suppress irrelevant background responses. The model is systematically evaluated on the Rice Leaf Bacterial and Fungal Disease (RLBF) dataset, captured in real rice fields under natural conditions. Additionally, a Rice-to-Corn cross-domain transfer experiment is conducted; PlantVillage-Corn is collected under relatively controlled conditions, so we treat this experiment as a complementary transfer sanity check rather than evidence of universal field-level robustness. Key metrics, including classification accuracy, F1-score, parameters (Params), FLOPs, and GPU-side inference latency, are reported for comparisons with baselines, ablation studies, and Grad-CAM++ based interpretability analysis.

Based on the above design and evaluation, the main contributions of this paper are summarized as follows:

- Incremental Lightweight Improvement: We build on GhostNetV3 and propose I-GhostNetV3 to improve fine-grained lesion representation in field imagery, achieving a favorable accuracy–efficiency trade-off while remaining compact relative to the original GhostNetV3 and targeting practical deployment on mobile/edge vision sensors.
- Two Plug-in Modules with Clear Motivation: We introduce APA and FCCA as pluggable attention modules with controlled overhead. APA emphasizes boundary-sensitive lesion cues via edge-guided parallel enhancement and gated fusion, and is selectively inserted at a few stage outputs; FCCA serves as a near-neutral SE replacement by coupling coordinate and efficient channel attention with lightweight fusion to reduce background distraction.
- Efficiency-Oriented Integration: We refactor the GhostNetV3 backbone in a modular manner and provide flexible insertion interfaces for APA/FCCA across stages. The backbone reconfiguration yields the primary efficiency gain, while the selective use of APA incurs additional computation to improve accuracy, resulting in a controlled and measurable accuracy–complexity trade-off verified by Params/FLOPs/latency reporting and ablation studies.
- Comprehensive Evaluation: We conduct experiments on the RLBF field dataset and a Rice-to-Corn cross-domain setting, including comparisons with multiple lightweight baselines, detailed ablations, and Grad-CAM++ visualizations to support interpretability and prospective practical deployment.

The remainder of this paper is structured as follows: Section 2 details the materials and methods. Specifically, the materials section provides the dataset sources and preprocessing details, while the methods section offers a comprehensive overview, including the I-GhostNetV3 algorithm, the APA and FCCA modules, and training details. Section 3 presents the results analysis. Section 4 contains the discussion. Section 5 summarizes the work and outlines future research directions.

## 2. Materials and Methods

### 2.1. Rice Disease Dataset

#### 2.1.1. Bacterial and Fungal Leaf Diseases of Rice Dataset

This study utilizes the Rice Leaf Bacterial and Fungal Disease (RLBF) dataset, which is publicly released on the Mendeley Data platform and widely used as a benchmark for rice disease recognition [18]. The dataset comprises 1701 raw RGB images of rice leaves across eight categories: Bacterial Leaf Blight, Brown Spot, Leaf Scald, Narrow Brown Leaf Spot, Rice Hispa, Sheath Blight, Leaf Blast, and Healthy Rice Leaf. According to the original dataset description, the images were captured between July and October 2023 at multiple locations in Sirajganj and Pabna Zilla, Bangladesh, during the local rice growing season, which is characterized by a warm and humid monsoon climate. The images were primarily acquired in real paddy fields using handheld RGB cameras under diverse outdoor lighting conditions and largely non-controlled backgrounds that often include soil, weeds, and water surfaces. As a result, the dataset exhibits substantial variation in illumination, background appearance, leaf pose, and lesion characteristics, such as large and small spots, irregular blotches, and both sharp and blurred lesion boundaries. These properties make RLBF particularly suitable for evaluating lightweight disease recognition models under visually complex field-like conditions.We note that RLBF is geographically limited and relatively small in scale; thus, robustness conclusions should be interpreted within this field-like benchmark, and broader real-field validation remains future work.

In this work, RLBF is chosen as the primary rice leaf disease dataset because it provides eight distinct classes with diverse lesion types in realistic scenes while remaining of a manageable size for detailed ablation studies and hyperparameter analysis. Although several additional rice disease datasets are available on public platforms (e.g., Kaggle), many of them mainly contain laboratory- or greenhouse-captured images with relatively clean and homogeneous backgrounds and partially overlapping disease categories with RLBF. Integrating multiple heterogeneous rice datasets into a unified and carefully controlled benchmark would require substantial additional effort in data harmonization and experimental design and is therefore beyond the scope of this paper. Accordingly, we do not claim broad geographic generalization; validation on independent field datasets collected from other regions/devices is left for future work. A more comprehensive multi-dataset evaluation across different rice disease datasets is left as an important direction for future research.

The eight rice leaf categories in the RLBF dataset exhibit distinct lesion characteristics in terms of distribution, size, shape, and color. Bacterial Leaf Blight typically shows yellowed leaf tips with elongated brown to whitish streaks extending along the blade. Brown Spot presents round brown spots with darker margins, whereas Healthy Rice Leaf has uniformly green tissue without visible lesions. Leaf Blast exhibits spindle-shaped lesions with gray centers and dark borders. Leaf Scald forms long straw-colored streaks with scorched tips. Narrow Brown Leaf Spot appears as very narrow dark-brown streaks distributed along the veins. Rice Hispa causes whitish scraped streaks parallel to the veins, and Sheath Blight produces gray-white patches with brown margins on sheaths and leaves.

**Figure 1:**
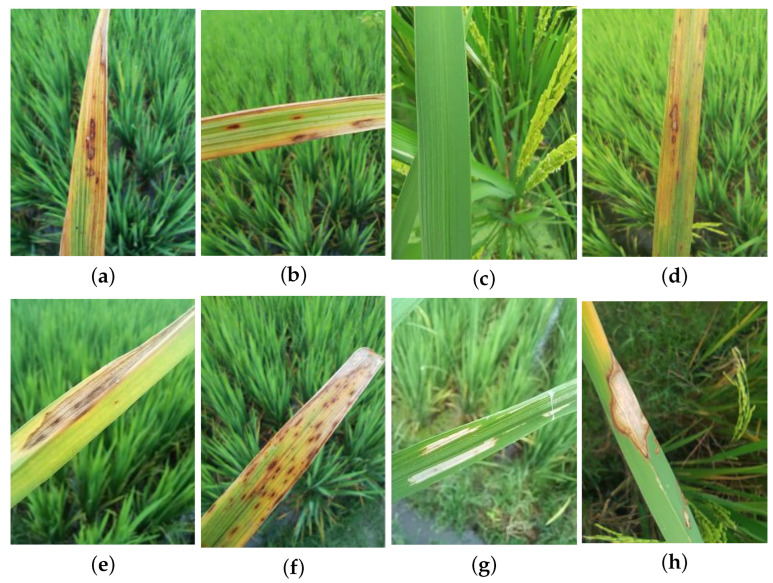
Sample images of eight rice leaf diseases: (**a**) Bacterial Leaf Blight, (**b**) Brown Spot, (**c**) Healthy Rice Leaf, (**d**) Leaf Blast, (**e**) Leaf Scald, (**f**) Narrow Brown Leaf Spot, (**g**) Rice Hispa, and (**h**) Sheath Blight.

Sample sizes and data partitioning by category are detailed in Table 1, and representative examples of each disease category are shown in Figure 1. To ensure fairness and reproducibility, the dataset is stratified by category and divided into training, validation, and test sets with an 8:1:1 ratio. All comparative methods in this study are evaluated on the same partitions.

#### 2.1.2. Vision Sensing Scenario in Smart Rice Farming

**Figure 2:**
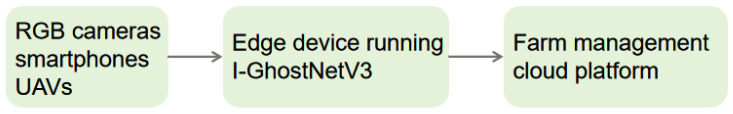
Conceptual deployment of I-GhostNetV3 with RGB vision sensors and edge devices in a smart rice farming system.

Although RLBF is a publicly available dataset, its RGB field images can be regarded as typical outputs of vision sensors deployed in smart rice farming. In practice, leaf images are often acquired by hand-held digital cameras, mobile phones, or UAV-mounted RGB cameras under natural illumination to monitor crop health without destructive sampling. Figure 2 illustrates a conceptual pipeline for deploying the proposed I-GhostNetV3 in a smart agriculture system based on vision sensors. First, RGB cameras or smartphones in the field periodically capture leaf images in rice paddies. Next, the lightweight I-GhostNetV3 model runs on a nearby edge device, such as a mobile phone, embedded GPU module, or IoT gateway, to perform real-time disease classification. Finally, the predicted results are transmitted to a farm management platform or cloud service, where they can be integrated with other agronomic information to support precise pesticide spraying, early warning of disease outbreaks, and informed decision-making.

#### 2.1.3. PlantVillage-Corn Dataset

To validate the cross-domain transfer performance of the proposed model, this study conducted a Rice-to-Corn transfer learning experiment using the Rice Leaf Disease dataset as the source domain and the PlantVillage-Corn dataset as the target domain. The PlantVillage-Corn dataset, sourced from the publicly available PlantVillage platform [19], comprises 3852 corn leaf images covering three typical diseases and healthy leaves. Compared to the Rice Leaf Bacterial and Fungal Disease dataset, images in the PlantVillage-Corn dataset were predominantly captured in relatively controlled laboratory or greenhouse environments. These images exhibit simpler backgrounds, more stable lighting conditions, and lower noise levels, making them closer to a “standardized” disease recognition scenario. However, it is important to note that the controlled conditions of the PlantVillage-Corn dataset differ significantly from the real-world field conditions encountered in agricultural environments. The lack of background complexity and natural environmental variations limits the dataset’s ability to fully represent the challenges faced in real-world disease recognition tasks. In addition, this Rice-to-Corn setting corresponds to transferring from a relatively complex field domain (RLBF) to a simpler, controlled domain (PlantVillage-Corn), which is an easier transfer direction than adapting models from standardized laboratory images to truly complex field scenes.

In the transfer experiments, we first pre-trained each model on the source domain Rice dataset, then fine-tuned and evaluated them on the target domain PlantVillage-Corn dataset using identical training configurations. The dataset was partitioned equally into training, validation, and test sets at an 8:1:1 ratio to ensure fairness in evaluating transfer performance. It should be noted that while the use of the PlantVillage-Corn dataset provides valuable insights into cross-domain transfer learning, its laboratory-based images do not fully validate the model’s generalization ability to field conditions. Therefore, the results should be viewed as an initial validation of transfer performance across different crops and scenarios, rather than a definitive test of generalization.

By establishing the target domain, we systematically analyzed the feature transfer and adaptation capabilities of I-GhostNetV3 and its comparison models across different crops and scenarios in disease recognition tasks.

#### 2.1.4. Field Validation as Future Work

Currently, due to the lack of sufficient real-world field data, field validation remains a key focus for our future research. Although this study uses the RLBF dataset, which indeed comes from real field environments and covers complex backgrounds and diverse lesion types, the dataset’s small scale and limited environmental variations mean that the current experiments do not fully represent all possible field conditions. In our current cross-domain experiments, we used the PlantVillage-Corn dataset for preliminary validation. However, this dataset mainly comes from a laboratory environment, with simpler backgrounds and more stable lighting conditions, and thus provides only limited evidence of the model’s robustness and generalization under truly complex and variable field conditions.

As a result, actual field validation will be a key focus of our future research. We plan to collect data from different field environments, which will include more variable lighting conditions, complex backgrounds (such as weeds, soil, and water puddles), and more challenging lesion types. With these real-world field data, we will further evaluate the performance of the I-GhostNetV3 model in actual agricultural environments, validate the model’s generalization ability, and improve the model to address more complex environmental challenges.

### 2.2. Image Preprocessing

Prior to model training, we performed uniform preprocessing on all input images. The raw RGB images, initially captured at a resolution of 1600 × 1200 pixels, were scaled proportionally and cropped to 224 × 224 pixels to match the input resolution used in all compared lightweight models. Pixel values were normalized to the [0, 1] range, and each channel was standardized using common settings for ImageNet pre-trained models (mean: (0.485, 0.456, 0.406), standard deviation: (0.229, 0.224, 0.225)).

Given the complexity of the RLBF dataset, which includes diverse lesion types and backgrounds, several data augmentation techniques were applied to the training set to increase sample diversity and mitigate overfitting risks. These augmentation strategies included random horizontal flipping (with a probability of 0.5), random rotation within a range of −30° to 30°, random cropping and scaling (within a ratio range of 0.8 to 1.0), and color jittering (affecting brightness, contrast, saturation, and hue). These operations simulate variations in lighting conditions, shooting angles, leaf orientations, and background clutter, improving the model’s robustness to challenges. Hybrid augmentations like Mixup or CutMix were also introduced in some experiments to further enhance the model’s robustness, especially to complex backgrounds and small lesions.

For the validation and test sets, only resizing and normalization were applied, ensuring consistency and fairness during evaluation. These preprocessing and augmentation steps allowed the experimental data to retain the complex features of the original field images while expanding the sample space, ultimately enhancing the generalization performance of I-GhostNetV3 and the comparison models in challenging environments.

### 2.3. Modeling Methods

#### 2.3.1. I-GhostNetV3 Network Architecture

**Figure 3:**
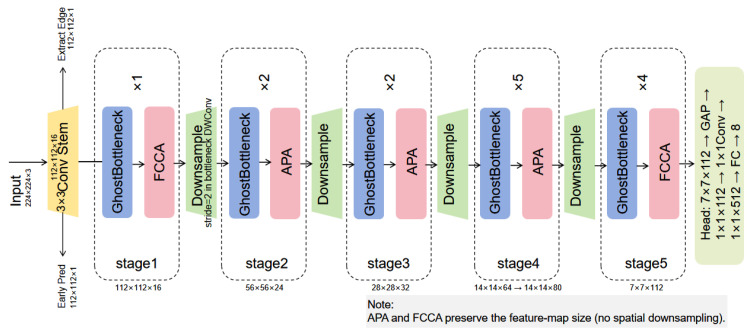
Schematic diagram of the proposed I-GhostNetV3 architecture.

This paper proposes an improved lightweight network architecture, I-GhostNetV3, built upon GhostNetV3 as the backbone. The goal is to enhance classification accuracy and generalization for rice leaf disease recognition from field RGB images while further reducing model parameters and computational overhead. As shown in Figure 3, the improvements focus on three key aspects. First, to further enhance the robustness of a GhostNetV3-based backbone to background noise and cross-domain distribution shifts in fine-grained lesion detection, we introduce the APA module, which combines an edge-guided spatial branch with an efficient channel attention branch to suppress irrelevant background responses and emphasize discriminative lesion features. Inspired by EGA [20] and ECA [21], APA integrates shallow edge features with deep semantic features to jointly model spatial location and channel information, thereby improving robustness to small lesions and blurred boundaries in complex backgrounds. Second, an FCCA module is designed to fuse coordinate-aware spatial features with channel responses. FCCA combines the spatial perception capability of CA [22] with the efficient channel modeling ability of ECA, and uses learnable fusion weights to adaptively balance these two components. While maintaining a lightweight design, this module strengthens the joint spatial–channel representation of the network, leading to more stable performance under large illumination variations and cluttered field scenes. Finally, the GhostNetV3 backbone is further optimized by streamlining the classification head and adjusting intermediate configurations. These modifications reduce redundant computation while enhancing feature representation and discriminative power, so that I-GhostNetV3 preserves the lightweight nature of GhostNetV3 and improves its ability to extract and classify fine-grained disease features.

#### 2.3.2. Overall Structure and Stage Division

I-GhostNetV3 adopts GhostNetV3 [23] as its backbone. As illustrated in Figure 3, the network consists of a convolutional stem followed by five feature extraction stages and a classification head. The input RGB image is first processed by a 3×3 convolution and non-linear activation to obtain initial low-level features. These features are then fed sequentially through Stages 1–5, where the spatial resolution is gradually reduced and the number of channels is increased to capture multi-scale semantic information. After the final stage, global average pooling and a 1×1 convolution compress the representation into a 512-dimensional feature vector, which is passed to a fully connected layer to produce predictions for eight rice disease categories.

On top of this backbone, I-GhostNetV3 embeds two types of attention modules within the core GhostBottleneck architecture. First, edge and guidance branches are constructed from shallow features output by the stem to extract edge maps and coarse prediction maps. APA modules leverage these shallow edge cues to guide and reweight deep features, highlighting lesion regions while suppressing interference from complex backgrounds; due to its noticeable overhead, APA is selectively inserted into only a few stage outputs where boundary-sensitive cues are most needed. Second, in mid-to-deep GhostBottleneck blocks, the FCCA module replaces the traditional SE structure [24], jointly modeling spatial position information and channel-wise responses to improve the characterization of small, low-contrast lesions. Overall, the backbone reconfiguration provides the primary efficiency gain, while the full I-GhostNetV3 intentionally introduces additional computation (mainly from selectively inserted APA) to obtain accuracy improvements; thus, the model should be viewed as an accuracy-enhanced lightweight variant that remains compact relative to the original GhostNetV3.

#### 2.3.3. APA Module

To improve robustness under field conditions, we introduce an APA module as an incremental enhancement on top of GhostNetV3 to compensate for two practical gaps: (i) repeated downsampling in compact backbones weakens boundary cues of small lesions, and (ii) purely channel-wise reweighting is insufficient to recover spatially coherent lesion structures in cluttered backgrounds. APA therefore combines spatial guidance and channel recalibration in a complementary manner through gated fusion, rather than proposing a fundamentally new attention paradigm. Since APA incurs noticeable computation, it is selectively inserted at only a few stage outputs under a controlled compute budget.

APA consists of two parallel branches. The first is a spatial guidance branch [20], which takes backbone features as input and incorporates an edge map and an early prediction map extracted from shallow features. By injecting these low-level priors, the branch highlights lesion-related boundaries and suppresses background textures, which is particularly beneficial when lesion saliency is weakened by aggressive downsampling.

The second is a channel attention branch [21], which obtains channel descriptors through global average pooling and employs one-dimensional convolutions to model local channel dependencies, adaptively emphasizing informative channels while suppressing redundant responses.

Compared with standard channel/spatial attention (e.g., SE/ECA or CBAM-style sequential attention), APA explicitly introduces edge- and prediction-related cues as shallow spatial priors and performs channel-wise gated fusion with complementary weights, enabling a controllable trade-off between boundary-sensitive spatial enhancement and discriminative channel recalibration.

Notation and tensor shapes: For simplicity, we omit the batch dimension *N* in the notation; all tensors are defined per sample. Let $X\in R^{C\times H\times W}$ be the input feature map. GAP(·) denotes global average pooling over (H,W), yielding $g=\mathrm{GAP}\left(X\right)\in R^{C\times 1\times 1}$, where $g_{c}=\frac{1}{HW}\sum_{i=1}^{H}\sum_{j=1}^{W}X_{c,i,j}$. σ(·) is the sigmoid function and ⊙ denotes element-wise multiplication. We follow the standard broadcasting rule: a tensor of shape C×1×1 is expanded to C×H×W by repeating values along (H,W) for channel-wise gating.

Given *X*, the spatial guidance branch (EGA) [20] enhances lesion boundaries using an edge map $E\in R^{1\times H_{e}\times W_{e}}$ and an early prediction map $P\in R^{1\times H_{p}\times W_{p}}$, which are resized to $\hat{E},\hat{P}\in R^{1\times H\times W}$ when necessary, producing a spatially enhanced feature $F_{s}\in R^{C\times H\times W}$. In parallel, the channel attention branch (ECA) [21] performs channel reweighting on *X* to obtain $F_{c}\in R^{C\times H\times W}$.

APA then applies a channel-wise gated fusion. Specifically, the gate is predicted from global context and constrained to be complementary:

(1) $$g=\mathrm{GAP}\left(X\right)\in R^{C\times 1\times 1},\;\omega_{s}=\sigma \left({\mathrm{Conv}}_{1\times 1}\left(g\right)\right)\in {\left(0,1\right)}^{C\times 1\times 1},\;\omega_{c}=1-\omega_{s},$$

where $1\in R^{C\times 1\times 1}$ is an all-ones tensor. Here, ${\mathrm{Conv}}_{1\times 1}$ is a pointwise convolution (equivalently a linear map with weight $W\in R^{C\times C}$) applied to $g\in R^{C\times 1\times 1}$, producing a gate in $R^{C\times 1\times 1}$. The gates are applied in a *channel-wise* manner by broadcasting along (H,W), i.e., ${\left(\omega_{s}⊙F_{s}\right)}_{c,i,j}=\omega_{s,c}\;F_{s,c,i,j}$.

Both branches preserve the feature shape, i.e., $F_{s},F_{c}\in R^{C\times H\times W}$. The fused feature is computed by

(2) $$F=\omega_{s}⊙F_{s}+\omega_{c}⊙F_{c}.$$

To verify that the fusion does not degenerate to a single branch, we summarize the learned gate $\omega_{s}$ on the validation set. Across all APA insertion points, the mean of $\omega_{s}$ is 0.500 (variance 0.023), and the saturation ratios $\mathrm{Pr}(\omega_{s}>0.9)$ and $\mathrm{Pr}(\omega_{s}<0.1)$ are 0.41% and 0.44%, respectively, indicating no collapse.

**Figure 4:**
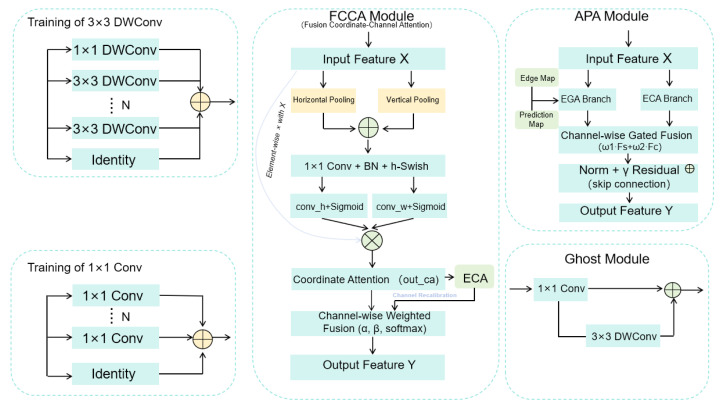
Internal architectures of the Ghost module, APA module, and FCCA module used in I-GhostNetV3.

The output is then obtained via a normalized residual form:

(3) Y=X+γ·Norm(F),

where Norm(·) denotes batch normalization (applied independently per channel, using statistics over the mini-batch and spatial locations), and γ∈R is a learnable scalar for residual scaling. As illustrated in Figure 4, APA is inserted after selected GhostNetV3 stage outputs and preserves the feature-map size (C×H×W→C×H×W), ensuring drop-in compatibility. The computational overhead is quantified in the table presented in the "Model Complexity and Efficiency" section; as a deliberate accuracy–efficiency trade-off, APA is applied only at a few stages to inject boundary-sensitive cues under a controlled compute budget.

#### 2.3.4. FCCA Module

To improve spatial–channel interaction under cluttered field backgrounds while keeping the attention overhead small, we design FCCA as an incremental drop-in replacement of the SE unit in GhostBottleneck blocks. Unlike SE/ECA, which mainly perform channel-wise recalibration, FCCA introduces axis-aware gating (Coordinate Attention, CA) [22] to encode long-range dependencies along the horizontal/vertical directions, which is beneficial when lesion patterns are spatially structured but surrounded by background clutter. Meanwhile, Efficient Channel Attention (ECA) [21] is retained for low-cost channel dependency modeling. Importantly, FCCA does not simply stack attention modules; it keeps both CA-gated and CA + ECA-gated features and performs a per-channel soft fusion to balance positional gating and channel recalibration.

Notation and tensor shapes: For simplicity, we omit the batch dimension *N*; all tensors are defined per sample. Let $X\in R^{C\times H\times W}$ be the input feature map. GAP(·) denotes global average pooling over (H,W), yielding $\mathrm{GAP}\left(X\right)\in R^{C\times 1\times 1}$. ⊙ denotes element-wise multiplication with standard broadcasting; e.g., ${\left(\alpha ⊙X\right)}_{c,i,j}=\alpha_{c}\;X_{c,i,j}$ for $\alpha \in R^{C\times 1\times 1}$.

Given an input feature map $X\in R^{C\times H\times W}$, FCCA first applies CA [22] to generate axis-aware gating weights $a_{h}\in R^{C\times H\times 1}$ and $a_{w}\in R^{C\times 1\times W}$, yielding

(4) $$X_{ca}=X⊙a_{h}⊙a_{w},$$

where $a_{h}$ is broadcast along the width dimension and $a_{w}$ is broadcast along the height dimension, so that $X_{ca}\in R^{C\times H\times W}$. Then, ECA [21] is applied to $X_{ca}$ to obtain a channel weight vector $s\in {\left(0,1\right)}^{C\times 1\times 1}$ and

(5) $$X_{eca}=X_{ca}⊙s.$$

To avoid over-suppressing fine lesion textures by a single attention path, FCCA performs a per-channel soft fusion. Specifically, we predict a 2C-dimensional gating tensor from global context,

(6) $$g={\mathrm{Conv}}_{1\times 1}\left(\mathrm{GAP}\left(X\right)\right)\in R^{2C\times 1\times 1},$$

split it along the channel dimension as $g=[g_{\alpha },g_{\beta }]$ with $g_{\alpha },g_{\beta }\in R^{C\times 1\times 1}$, and apply a 2-way softmax per channel:

(7) $$\left[\alpha_{c},\beta_{c}\right]=\mathrm{softmax}\left([g_{\alpha ,c},g_{\beta ,c}]\right),\;\forall c\in \left\{1,\ldots ,C\right\},\;Y=\alpha ⊙X_{ca}+\beta ⊙X_{eca},$$

where $\alpha ,\beta \in R^{C\times 1\times 1}$ and $\alpha_{c}+\beta_{c}=1$ for each channel *c*. Compared with a direct CA→ECA cascade, the proposed soft fusion explicitly retains the CA-only path and adaptively interpolates it with the CA + ECA path on a per-channel basis, improving robustness to background clutter without introducing extra branches or heavy convolutions.

FCCA preserves the tensor shape (C×H×W→C×H×W) and serves as a drop-in replacement of the original SE unit inside GhostBottleneck blocks (Figure 4), requiring no changes to the shortcut connection. Its overhead is negligible relative to SE, making it suitable as the default enhancement throughout the backbone. In our design, FCCA is applied broadly to stabilize spatial–channel interactions in mid-to-deep stages, while APA is used sparingly at selected stage outputs when boundary-sensitive spatial cues are required under a higher compute budget.

#### 2.3.5. Positioning of APA and FCCA

Table 2 positions APA and FCCA against representative attention blocks along five practical dimensions (cues, guidance, fusion, deployment, and the addressed limitation in GhostNetV3).

This comparison highlights that APA is designed to inject lesion boundary cues via edge-guided spatial enhancement at selected stage outputs, complementing the channel recalibration ability of lightweight channel attention, whereas FCCA serves as a drop-in replacement for SE by softly fusing coordinate-aware and channel-wise cues with very small overhead relative to SE. Such positioning clarifies the technical motivation of the two modules and explains why they are inserted at different locations in GhostNetV3.

#### 2.3.6. Complexity Analysis

To quantitatively characterize the lightweight properties of the proposed I-GhostNetV3, we use the number of parameters (Params) and floating-point operations (FLOPs) as metrics for evaluating model complexity. The parameter count is obtained by calculating the total number of learnable parameters in the network, while FLOPs are estimated for each model using the same computational script under an input resolution of 224 × 224. Additionally, to reflect the model’s efficiency in practical deployment, single-image inference latency and throughput are measured.

### 2.4. Training Settings

All experiments were conducted under the same training environment and hyperparameter settings to ensure fair comparison. All models were implemented in Python 3.11.11 using the PyTorch 2.3.1 deep learning framework with CUDA 12.1, and trained and evaluated on a single NVIDIA GeForce RTX 3080 Ti GPU (NVIDIA Corporation, Santa Clara, CA, USA). Latency and throughput are measured on the same RTX 3080 Ti setup (batch size = 1) and reported as GPU-side proxies for relative comparison, rather than on-device measurements on edge hardware (e.g., Jetson/mobile SoCs). The Rice Leaf Bacterial and Fungal Disease (RLBF) dataset was used in all experiments. Input images were resized to 224×224 pixels and normalized with mean values of (0.485, 0.456, 0.406) and standard deviations of (0.229, 0.224, 0.225).

For the baseline models, we adopted the AdamW optimizer with an initial learning rate of 0.0015, a weight decay of $2\times 10^{-4}$, a batch size of 32, and 150 training epochs. To mitigate overfitting, regularization included dropout together with basic data augmentation strategies such as random rotation, horizontal flipping, and color perturbation. The cosine annealing learning rate scheduler was used to adjust the learning rate, allowing it to gradually decay following a cosine curve. This helps the model converge more smoothly and avoids sudden changes in learning rate, enhancing training stability.

To ensure a fair comparison, we applied an identical augmentation pipeline to all models, including basic augmentations (random rotation, horizontal flipping, and color perturbation) as well as Mixup and CutMix with the same hyperparameters (Mixup = 0.1, CutMix = 0.3). Mixed-precision training (AMP) was enabled for all models under the same setting to improve GPU-side training efficiency while maintaining numerical stability. To assess run-to-run variability and ensure reproducibility, each experiment was repeated seven times with different random seeds (0–6). For each run, the chosen seed was consistently applied to Python, NumPy (v1.26.4), and PyTorch with CUDA (v12.6). Furthermore, we used multi-threaded data loading with num_workers = 6 to accelerate data I/O and reduce bottlenecks during training, and gradient clipping was employed to avoid potential gradient explosion and yield smoother parameter updates. All other hyperparameter configurations were kept consistent with the baseline models. The experimental settings are summarized in Table 3.

### 2.5. Evaluation Metrics

To evaluate the performance of the models in classifying rice leaf diseases, we use precision (*P*), recall (*R*), accuracy (Acc), and F1-score ($F_{1}$). They are defined as

(8) $$\begin{array}{cc} P & =\frac{TP}{TP+FP} \end{array}$$

(9) $$\begin{array}{cc} R & =\frac{TP}{TP+FN} \end{array}$$

(10) $$\begin{array}{cc} \mathrm{Acc} & =\frac{TP+TN}{TP+FP+TN+FN} \end{array}$$

(11) $$\begin{array}{cc} F_{1} & =\frac{2PR}{P+R} \end{array}$$

Multi-class setting: Our task is single-label multi-class classification with *K* disease classes. We compute precision/recall/$F_{1}$ using a one-vs-rest strategy: for each class *k*, $TP_{k},FP_{k},FN_{k}$ are counted by treating class *k* as positive and all other classes as negative. Then,

(12) $$P_{k}=\frac{TP_{k}}{TP_{k}+FP_{k}},\;R_{k}=\frac{TP_{k}}{TP_{k}+FN_{k}},\;F1_{k}=\frac{2P_{k}R_{k}}{P_{k}+R_{k}}.$$

Macro-averaged metrics are computed as $\frac{1}{K}\sum_{k=1}^{K}\left(\cdot \right)$, while weighted-averaged metrics weight each class by its support $n_{k}$: $\sum_{k=1}^{K}\frac{n_{k}}{N}\left(\cdot \right)$ with $N=\sum_{k}n_{k}$. Micro-averaged precision/recall/F1 are obtained by aggregating counts across classes: $TP=\sum_{k}TP_{k}$, $FP=\sum_{k}FP_{k}$, $FN=\sum_{k}FN_{k}$, and applying Equations (1)–(4). Overall accuracy is computed as $\mathrm{Acc}=\frac{1}{N}\sum_{i=1}^{N}I\left({\hat{y}}_{i}=y_{i}\right)$.

In these formulas, the terms are defined as follows:

In the multi-class case, $TP_{k}/FP_{k}/FN_{k}$ are defined per class under the one-vs-rest scheme described above (i.e., class *k* is treated as positive and others as negative).
TP (True Positive)—The number of samples that are correctly classified as positive by the model and are indeed positive.
TN (True Negative)—The number of samples that are correctly classified as negative by the model and are indeed negative.
FP (False Positive)—The number of samples that are incorrectly classified as positive by the model but are actually negative.
FN (False Negative)—The number of samples that are incorrectly classified as negative by the model but are actually positive.
*P* (Precision)—The proportion of samples predicted as positive that are truly positive.
*R* (Recall)—The proportion of actual positive samples that are correctly classified as positive.
Acc (Accuracy)—The proportion of all samples that are correctly classified by the model, reflecting its overall performance.
$F_{1}$ (F1-score)—A harmonic mean of precision and recall; a higher $F_{1}$ indicates better balance between them.

## 3. Results

### 3.1. Overall Performance

To comprehensively evaluate the effectiveness of the proposed I-GhostNetV3, we compare it with representative lightweight models and strong high-capacity CNN baselines. The baselines include classic MobileNetV2 [26], MobileNetV3-Large [27], MobileNetV3-Small [27], EfficientNet-B0 [28], InceptionV3 [29], and DenseNet-121 [30], as well as some recent lightweight architectures such as MobileNetV4-Conv-S [31] and RepViT-M1.0 [32].

We use accuracy, precision, recall, and F1-score as the primary evaluation metrics, while also reporting FLOPs, Params, latency, and throughput to analyze the trade-off between performance and efficiency. To account for randomness introduced by parameter initialization and data shuffling, each model is trained seven times with different random seeds, and all accuracy, precision, recall, and F1-score values in Table 4 are reported as the mean ± 95% confidence interval over these runs. Std (Acc) denotes the standard deviation of accuracy across runs. In addition, we apply McNemar’s test on paired per-sample predictions to assess statistical significance for selected model comparisons (Table 5).

As shown in Table 4, I-GhostNetV3 achieves the highest *mean* Top-1 accuracy of 90.02%±0.12 among the compared models on the RLBF dataset, showing notable gains over several widely used lightweight CNN baselines such as MobileNetV2-100 (86.39%±0.26) and EfficientNet-B0 (86.78%±0.48). It also improves over several lightweight baselines in terms of precision, recall, and F1-score, suggesting that the backbone reconfiguration together with APA and FCCA enhances lesion-related feature discrimination under complex field backgrounds.

We additionally conduct McNemar’s test on paired per-sample predictions over the same RLBF test set (Table 5). Here, $n_{10}$ denotes the number of samples misclassified by the baseline but correctly classified by I-GhostNetV3, and $n_{01}$ denotes the opposite case. With continuity correction (df=1), I-GhostNetV3 shows statistically significant differences against GhostNetV3 (1.3×) and MobileNetV2-100 (p<0.05). In contrast, the difference compared with a strong high-capacity baseline (InceptionV3) is not significant (p=0.499), indicating statistically indistinguishable performance on this dataset at α=0.05. Accordingly, we interpret the results as significant improvements over selected lightweight baselines, while being comparable to InceptionV3 under the current test set.

**Figure 5:**
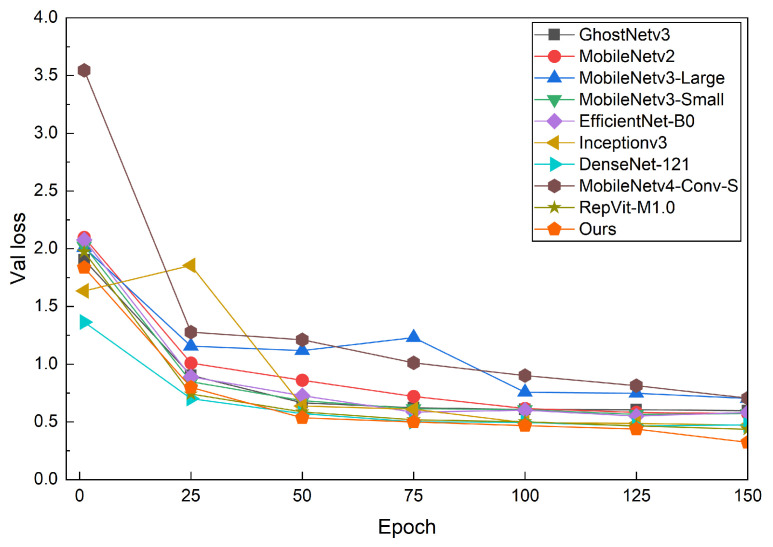
Validation loss curves on RLBF. I-GhostNetV3 converges to a lower loss and shows stable behavior in later epochs compared with the baseline lightweight CNNs.

**Figure 6:**
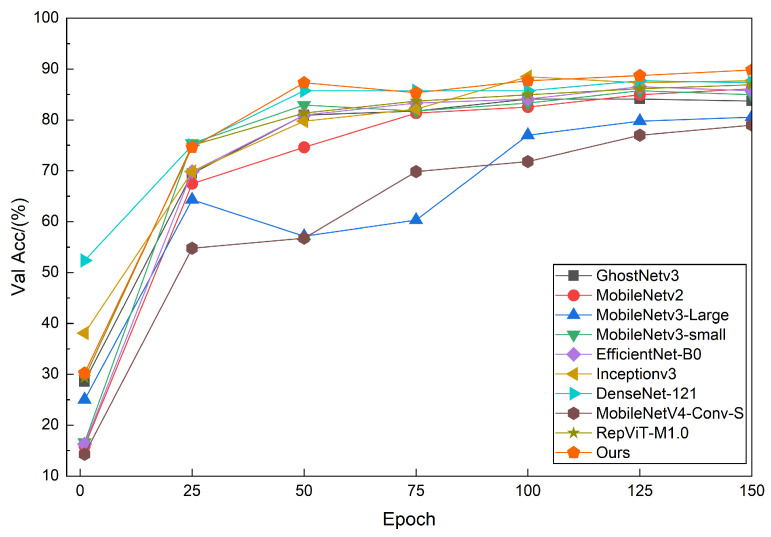
Validation accuracy curves on RLBF. I-GhostNetV3 achieves higher final validation accuracy among the compared models.

The validation loss and accuracy curves in Figure 5 and Figure 6 illustrate the convergence behavior of different models under a consistent evaluation protocol. We focus on validation curves to better reflect generalization behavior on unseen data, given the limited scale of RLBF. Specifically, I-GhostNetV3 starts from a loss level comparable to or slightly lower than the baseline models, but its validation loss decreases more rapidly and stabilizes earlier. In contrast, GhostNetV3, MobileNetV3-Large, and MobileNetV4-Conv-S exhibit higher initial loss values with larger oscillations in the early training stages, indicating less stable optimization. EfficientNet-B0 and RepViT-M1.0 converge more slowly and reach lower final accuracy than I-GhostNetV3. Overall, these curves suggest that the proposed backbone reconfiguration together with the APA and FCCA modules improves both convergence speed and training stability on the RLBF dataset. As all models are trained under identical optimization and augmentation settings, the validation curves provide a consistent basis for comparing generalization behavior across architectures.

We note that RLBF is a relatively small dataset and that strong data augmentation strategies are employed, which may introduce a risk of overfitting or optimistic performance estimates. To mitigate this issue, all models are evaluated under identical training protocols and trained seven times with different random seeds, with performance reported as mean ± 95% confidence intervals. In addition, paired statistical tests (McNemar’s test) are conducted to further assess the robustness and reliability of the observed performance differences.

### 3.2. Model Complexity and Efficiency

**Figure 7:**
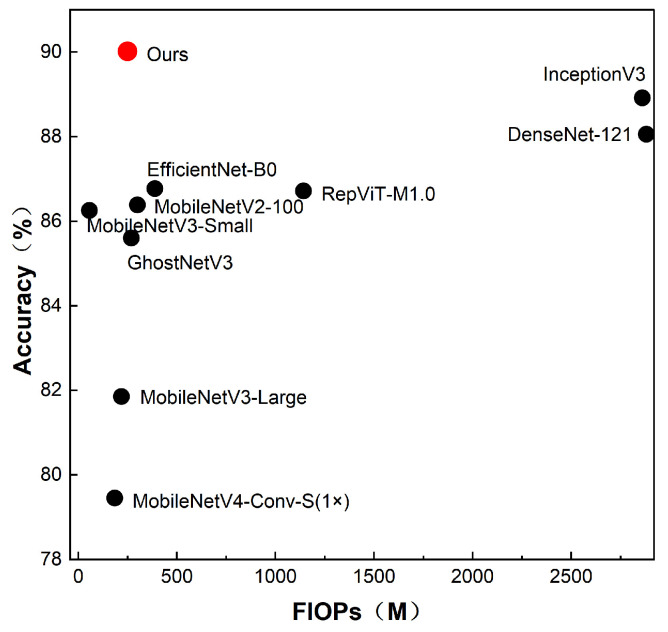
Top-1 accuracy versus FLOPs for I-GhostNetV3 and comparison models on the Rice Leaf Bacterial and Fungal Disease dataset.

To systematically evaluate the efficiency profile of I-GhostNetV3, we compare it with representative networks using Table 4 and Figure 7, focusing on parameter size, computational complexity, and GPU-side inference efficiency.

Parameter size: I-GhostNetV3 contains 1.831 million parameters, which is approximately 79.1% fewer than the 8.754 million parameters of the original GhostNetV3, and is also smaller than medium-capacity backbones such as EfficientNet-B0. Under this reference point (original GhostNetV3), the proposed model achieves a favorable accuracy–size trade-off. Meanwhile, it is important to clarify that the full I-GhostNetV3 is intentionally larger than the reconfigured backbone-only variant reported in the ablation study (Table 6), because additional modules (mainly selectively inserted APA) are introduced to improve accuracy. Therefore, the design should be viewed as an accuracy-enhanced variant built upon a reconfigured lightweight backbone, rather than a pure compression of the backbone-only configuration.

Computational complexity: I-GhostNetV3 requires 248.694 MFLOPs, which is about 55% of GhostNetV3’s FLOPs and falls in the range of widely used mobile backbones such as MobileNetV2/MobileNetV3-Large. As shown in Figure 7, I-GhostNetV3 achieves strong accuracy among lightweight CNN baselines under our experimental setup, suggesting a competitive accuracy–complexity trade-off. Consistent with the discussion above, compared with the backbone-only variant (Table 6), the full model increases FLOPs as a deliberate trade-off to obtain higher recognition accuracy.

Inference efficiency: On an NVIDIA GeForce RTX 3080 Ti GPU, I-GhostNetV3 achieves a single-image latency of 1.270 ms/image (787.76 img/s). Although it is slower than ultra-minimalist models such as MobileNetV2, it is substantially faster than medium-to-large networks (e.g., InceptionV3 and DenseNet-121) under the same GPU setting. These GPU-side measurements serve as a proxy for runtime efficiency and suggest that the model is a promising candidate for future edge deployment; however, on-device profiling on mobile/embedded hardware remains to be validated.

In summary, I-GhostNetV3 provides a favorable accuracy–efficiency trade-off for lightweight rice leaf disease recognition: it is substantially more compact than the original GhostNetV3, while the full model deliberately introduces additional computation over the backbone-only configuration to obtain accuracy gains. This supports its potential for future deployment on mobile and embedded platforms, pending on-device validation.

### 3.3. Ablation Study

To evaluate the contribution of each component, we conducted ablation experiments by progressively enabling backbone reconfiguration and different attention modules. The results on the Rice Leaf Bacterial and Fungal Disease dataset are summarized in Table 6. The original GhostNetV3 (1.3×) baseline achieves an accuracy of 85.53±0.24 with 8.754 M parameters and 269.000 MFLOPs. After applying the backbone reconfiguration, the number of parameters is reduced to 0.383 M and the computational cost drops to 59.555 MFLOPs, while the accuracy improves to 86.26±0.12. This indicates that the reconfigured backbone provides the primary efficiency gain and improves the accuracy–efficiency trade-off for field rice disease recognition.

On top of the reconfigured backbone, we evaluate two widely used attention baselines, ECA and CA, by replacing the original SE blocks. Replacing SE with ECA achieves 86.37±0.21 accuracy with very small overhead relative to SE (0.386 M parameters and 59.939 MFLOPs). Similarly, replacing SE with CA yields a comparable accuracy of 86.37±0.11 with slightly higher complexity (0.401 M, 60.716 MFLOPs). These results suggest that standard lightweight channel/coordinate attention baselines provide only limited gains under a controlled compute budget.

We then assess our spatial enhancement designs. Replacing SE with the proposed APA increases the accuracy to 87.70±0.12 with 0.548 M parameters and 85.970 MFLOPs. Compared with the backbone-only variant (with SE), this corresponds to an additional 26.415 MFLOPs (59.555 → 85.970), indicating that APA introduces a noticeable computational overhead. As quantified in Table 7, the edge-guided spatial branch accounts for 78.4% of the profiled MFLOPs within APA and contributes 24.1% of the total network MFLOPs in the Backbone + APA setting, suggesting that the increased complexity is dominated by the edge-guided spatial enhancement. To reduce the computational burden, we further evaluate an APA-lite variant (also used as an SE replacement). As shown in Table 6, APA-lite reduces the computation from 85.970 to 65.539 MFLOPs (a 23.8% reduction) with only a minor accuracy drop (87.70% → 87.45%). Moreover, the gain of APA is consistent across seven independent runs, and its 95% confidence interval is comparable to other attention baselines in Table 6, indicating that the improvement is stable across runs.

In contrast, replacing SE with FCCA yields 87.82±0.11% accuracy with very small overhead relative to SE in Table 6 (0.342 M parameters and 59.842 MFLOPs), indicating that FCCA can enhance coordinate–channel interaction while remaining close to the backbone-only setting. Finally, within the ablation settings in Table 6, the proposed I-GhostNetV3 achieves the highest accuracy by using FCCA as an SE replacement and additionally inserting APA (marked with ${✓}^{†}$ in Table 6), reaching 90.02±0.12% accuracy with 1.831 M parameters and 248.694 MFLOPs. Compared with the backbone-only model, this corresponds to an absolute accuracy gain of 3.76 percentage points (86.26% → 90.02%) at the cost of a clear increase in model size and FLOPs, reflecting a deliberate accuracy–efficiency trade-off. Nevertheless, the full model remains substantially more compact than the original GhostNetV3, and the results suggest that FCCA (small-overhead SE replacement) and selectively inserted APA are complementary for improving lesion-related representations under field conditions.

### 3.4. Cross-Domain Transfer Experiment

To evaluate the cross-domain transfer ability of the proposed model, we conducted a cross-domain transfer learning experiment from the Rice Leaf Bacterial and Fungal Disease (RLBF) dataset to the PlantVillage–Corn dataset. The models were initially trained to convergence on the RLBF dataset to obtain the optimal weights for the source domain. These weights were then transferred to the PlantVillage–Corn dataset for fine-tuning and evaluation under identical parameter configurations and optimization strategies.

Given the research focus on lightweight design and practical deployment efficiency, four representative lightweight models with distinct structural characteristics were selected for cross-domain evaluation to ensure fair and representative comparisons:

- GhostNetV3, serving as the baseline lightweight convolutional network;
- MobileNetV4-Conv-S (1×), representing the mainstream direction of current efficient convolutional architectures;
- RepViT-M1.0, a hybrid architecture combining convolutional and Transformer features;
- I-GhostNetV3 (Ours), the improved network proposed in this paper, integrating APA and FCCA modules.

Table 8 reports the Rice→Corn transfer results on PlantVillage–Corn. Overall, all models achieve high accuracy on this target domain, which is consistent with its relatively clean imaging conditions (homogeneous backgrounds and well-contrasted lesions). Note that this transfer direction is RLBF→PlantVillage (complex→simple), which is generally favorable; therefore, it should be interpreted only as a transfer sanity check. Among the compared methods, I-GhostNetV3 achieves the highest mean accuracy (Acc: 98.22%±0.16; F1: 97.89%±0.29), with a small margin over the original GhostNetV3 (Acc: 97.82%±0.21). The small gap suggests that APA/FCCA do not introduce noticeable degradation when adapting to a simpler controlled domain (where the benefit of enhanced lesion modeling can be less pronounced); additional validation on independent multi-site field datasets remains future work.

Future work will include more challenging multi-site field datasets to further assess robustness in real-field practical scenarios.

### 3.5. Confusion Matrix Analysis

**Figure 8:**
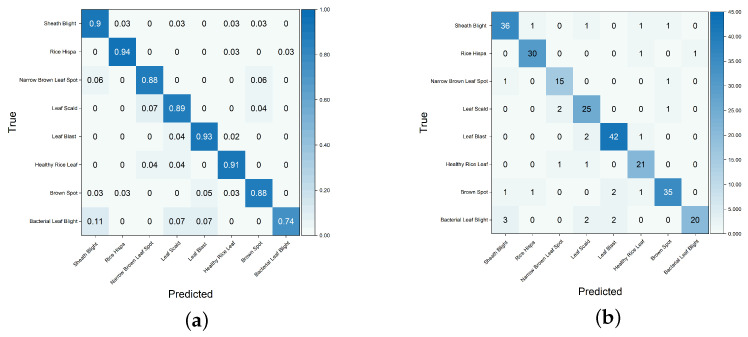
I-GhostNetV3 Confusion Matrix. (**a**) Confusion Matrix; (**b**) Normalized Confusion Matrix. The main diagonal of the confusion matrix (blue section) represents correct predictions, while the remaining sections indicate misclassifications.

To further evaluate the recognition performance of I-GhostNetV3 across different categories, Figure 8 depicts the confusion matrix of the model on the RLBF test set. Overall, the diagonal-dominant pattern indicates that most samples are correctly classified. Notably, the model achieves the highest recalls for “Rice Hispa” and “Leaf Blast”, reaching 0.9375 and 0.9333, respectively, suggesting that these categories can be reliably recognized under the current dataset setting. Table 9 further reports the per-class Precision/Recall/F1 scores, together with macro-, weighted-, and micro-averaged results derived from the same confusion matrix.

Despite the overall strong performance, the confusion matrix still reveals occasional misclassifications between visually similar disease categories, especially “Brown Spot” and “Narrow Brown Leaf Spot”. From an agronomic perspective, Brown Spot lesions are generally round or oval brown spots with relatively diffuse boundaries and, in many cases, a surrounding yellow halo, whereas Narrow Brown Leaf Spot typically presents as slender, elongated dark-brown streaks distributed along the leaf veins. In real field conditions, these morphological differences can become less evident at different growth stages or under varying environmental conditions (e.g., leaf aging, overlapping lesions, or lighting changes), causing the visual appearances of the two diseases in RGB images to become highly similar.

**Figure 9:**
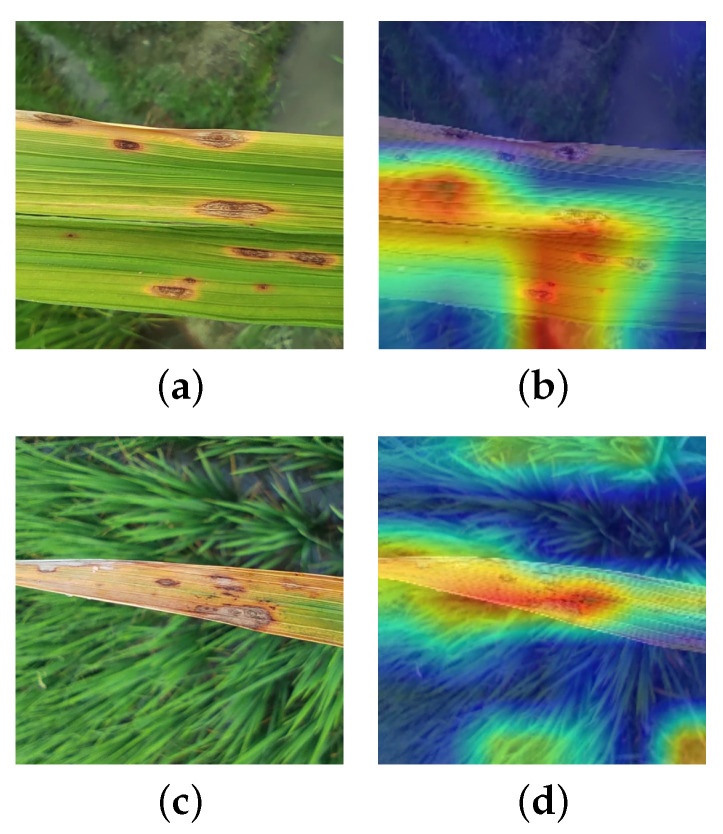
Grad-CAM++ visualizations for representative samples of Brown Spot and Narrow Brown Leaf Spot. (**a**) Brown Spot (original), (**b**) Brown Spot (Grad-CAM++), (**c**) Narrow Brown Leaf Spot (original), (**d**) Narrow Brown Leaf Spot (Grad-CAM++).

At the representation-learning level, we further inspected Grad-CAM++ feature maps from the last convolutional stage for representative samples of these two classes (see Figure 9). The activation regions of I-GhostNetV3 for both “Brown Spot” and “Narrow Brown Leaf Spot” concentrate on the brown necrotic areas along the leaf blade rather than on the background, and the highlighted patterns partially resemble each other when spots become elongated or merge along the veins. This similar attention distribution helps explain the occasional misclassifications between the two categories observed in the confusion matrix.

Moreover, in practical agricultural scenarios, multiple disease symptoms may coexist on the same leaf, while the RLBF dataset adopts a single-label annotation scheme at the image level. When co-occurring lesions include both round and elongated spots, assigning only one dominant label may further obscure the boundary between “Brown Spot” and “Narrow Brown Leaf Spot”. In future work, a multi-label learning framework with more detailed lesion-level annotation will be explored to better handle such mixed symptoms and to reduce misclassification between visually similar disease categories.

### 3.6. Visualization

To further examine how different models respond to lesion regions, we adopt Grad-CAM++ [33] for qualitative visualization. For each network, the heatmaps are computed from the last convolutional block before global average pooling, using the predicted class score as the target. All Grad-CAM++ maps are generated with the same implementation and settings, normalized to [0,1], resized to the input resolution, and overlaid on the original RGB images with a unified blue–red colormap, where warmer colors indicate higher contribution to the final prediction.

**Figure 10:**
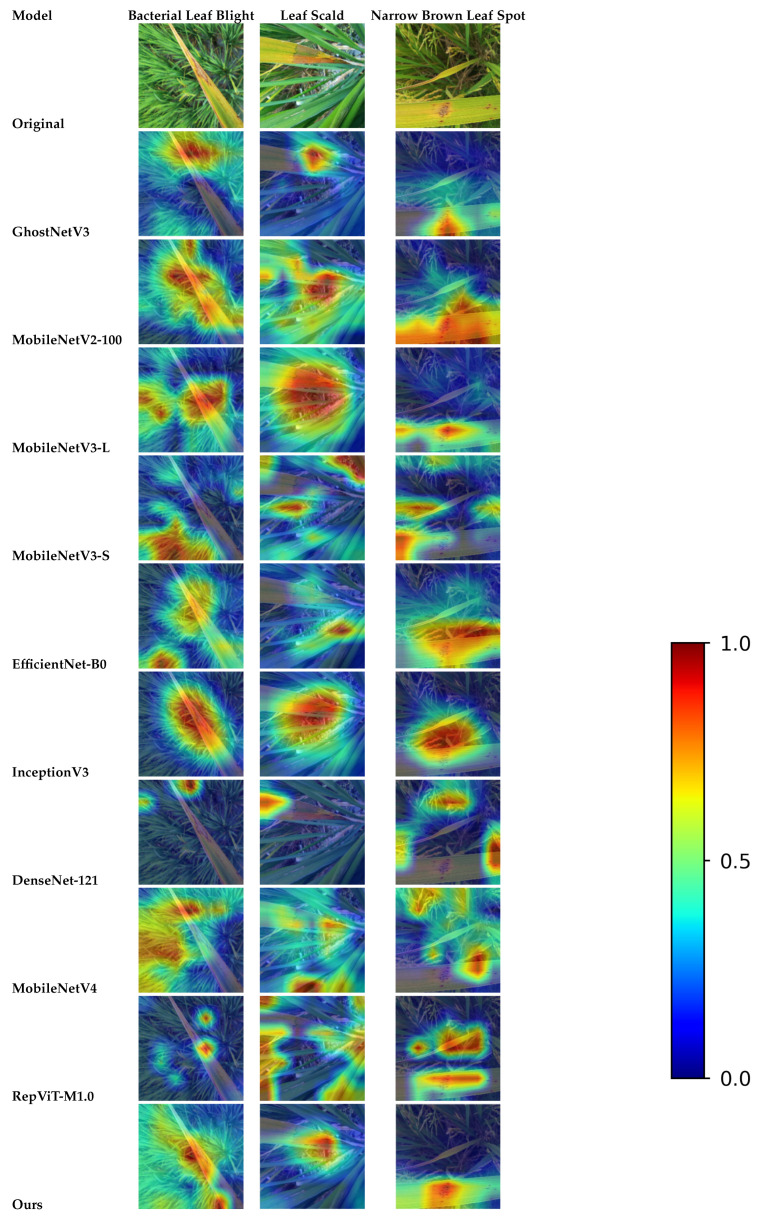
Grad-CAM++ visualizations of different models on representative rice disease categories. The Grad-CAM++ maps are min–max normalized to [0,1] and visualized with the JET colormap (red: high response, blue: low response).

Three representative rice leaf disease samples (Bacterial Leaf Blight, Leaf Scald, and Narrow Brown Leaf Spot) from the test set are selected for comparison. In Figure 10, each row corresponds to one model (listed in the leftmost column), and the three columns with black borders show the Grad-CAM++ results on these three images. Overall, the improved I-GhostNetV3 produces stronger and more compact responses within the true lesion areas, especially along lesion cores and boundaries. In contrast, GhostNetV3 and several other lightweight baselines often display more scattered activations and non-negligible responses on background structures such as leaf veins or non-diseased tissue. For example, in the Narrow Brown Leaf Spot sample, some baselines partially miss the elongated streak-like lesions or highlight adjacent veins, whereas I-GhostNetV3 yields continuous, lesion-centred activations along the diseased regions. Similar behaviour is observed for Bacterial Leaf Blight and Leaf Scald, where the proposed model suppresses irrelevant background clutter and better covers irregular lesion patches.

This behaviour is consistent with the design of the APA and FCCA modules: the edge-guided mechanism in APA encourages the network to emphasize lesion boundaries, while the channel recalibration in FCCA helps assign higher weights to disease-relevant feature channels and down-weight background-related responses. Together, these mechanisms lead to sharper and more lesion-focused activation patterns in I-GhostNetV3, which supports the interpretability of the proposed architectural modifications. A more systematic visualization of the internal APA/FCCA attention maps will be investigated in future work.

## 4. Discussion

In practical agricultural applications, accurately identifying rice leaf diseases remains highly challenging. On the one hand, there are pronounced inter-class similarities: for example, Brown Spot and Bacterial Leaf Blight may exhibit similar lesion structures and color changes in their early stages, making it difficult for models to establish clear decision boundaries at the feature level. On the other hand, there are substantial intra-class variations within the same disease category. As rice grows, disease progression advances, and management practices differ, lesion morphology, size, texture, and coloration continually change, requiring models to exhibit strong adaptability in feature representation. Furthermore, naturally captured field images are affected by various disturbances—including varying light intensity, complex backgrounds, leaf occlusion, and inconsistent shooting angles—which often introduce noise. Such noise can cause disease regions to appear as weak textures, blurred areas, or partially missing structures, significantly reducing the discriminative capability of traditional convolutional models [34,35]. Recent studies have attempted to address these challenges in real field environments. For instance, Li et al. developed RDRM-YOLO, a lightweight and high-accuracy detection model designed to improve robustness under complex outdoor conditions [36]. Therefore, achieving robust modeling of fine-grained lesion features in complex field environments remains a core challenge for automated rice disease identification.

Against this backdrop, the proposed I-GhostNetV3 addresses these challenges by modularly reconstructing the GhostNetV3 backbone and incorporating two attention mechanisms—APA and FCCA—to enhance the model’s ability to characterize lesion regions in complex scenarios. Unlike prior studies [37,38,39] that rely on a single attention module to improve performance, this work adopts a synergistic spatial–channel dual-attention design. Specifically, traditional CA enhances spatial feature encoding by capturing positional dependencies, but it is limited in modeling inter-channel relationships; ECA refines channel dependencies with minimal parameter overhead but lacks explicit spatial localization capability; EGA integrates broader contextual information to enhance global feature perception, but it remains insufficient for modeling fine-grained cross-dimensional feature interactions. The proposed APA module achieves complementary modeling of spatial and channel attention through parallel EGA and ECA branches. FCCA further introduces a channel recalibration mechanism on top of CA, enabling the model to focus simultaneously on local lesion details and global cross-channel consistency. Ablation experiments demonstrate that the synergistic interaction of these two attention mechanisms significantly enhances the ability to distinguish complex lesion textures and subtle differences, without introducing excessive parameter overhead.

**Figure 11:**
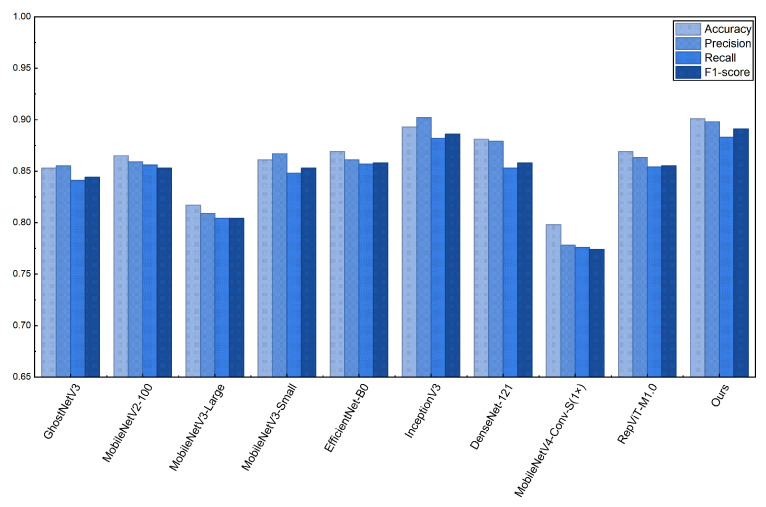
Performance Comparison of Different Models for Rice Disease Image Classification.

In terms of overall performance, I-GhostNetV3 achieves the highest mean accuracy on the constructed rice leaf disease image dataset and shows strong precision, recall, and F1-score among the evaluated models. Compared with the original GhostNetV3, it reduces parameters by approximately 79.1% (8.754 M → 1.831 M) while improving the overall accuracy–efficiency trade-off under our experimental setup. Compared with representative lightweight baselines (e.g., MobileNet and EfficientNet), I-GhostNetV3 demonstrates a competitive balance between model size, computational complexity, and classification accuracy. While the minimalist MobileNetV3-Small has the lowest FLOPs, it exhibits lower accuracy on RLBF, suggesting limited capacity under challenging field-like conditions. Conversely, some networks obtain higher accuracy by increasing width or depth, at the cost of substantially higher parameters and FLOPs. Table 4 and Figure 7 show that I-GhostNetV3 is close to the accuracy–complexity Pareto frontier among the compared models in this study. Figure 11 provides a more intuitive comparison of classification performance across models on RLBF. Overall, the results suggest that APA and FCCA strengthen lesion-related representations under lightweight constraints, making I-GhostNetV3 a promising candidate for future deployment on resource-constrained edge devices, pending hardware-level profiling and validation.

Grad-CAM++ visualization results further corroborate the above analysis. Compared with the baseline model, I-GhostNetV3 focuses more accurately on lesion regions rather than background noise or healthy leaf tissue across most samples. Notably, even in cases where lesions are small, texturally weak, or partially occluded, the attention heatmaps still cover the critical lesion regions. This demonstrates that the APA and FCCA modules not only enhance classification performance in terms of numerical metrics but also improve the model’s interpretability and practical application value from the perspective of spatial attention.

Although I-GhostNetV3 performs well in most disease identification tasks, certain limitations remain. First, the current experiments primarily rely on the Rice Leaf Bacterial and Fungal Disease Dataset, which is relatively small and lacks multi-crop and multi-scenario coverage. Future research should extend to disease datasets from other crops and more diverse environments to further validate the model’s generalization capability. Furthermore, when transferring the model to the PlantVillage-Corn dataset, the laboratory-collected nature of this dataset, with relatively clean backgrounds, may underestimate the difficulty of generalization in real-world environments compared with complex field scenarios. In addition, the model still exhibits misclassifications in highly similar categories (e.g., Brown Spot and Narrow Brown Leaf Spot), indicating room for improvement in capturing extremely subtle textural and morphological differences with the current feature representation. To address these issues, future work could enhance the model’s ability to distinguish highly similar categories by integrating mechanisms such as multi-scale feature fusion, semantic priors, or graph-structure modeling. Moreover, incorporating learning strategies such as semi-supervised learning, self-supervised learning, and domain generalization could reduce reliance on large-scale fine-grained annotations and further improve the model’s robustness and generalization in complex, uncontrolled scenarios.

Beyond its technical performance, the proposed I-GhostNetV3 also provides meaningful agricultural value. Accurate and timely identification of rice leaf diseases is crucial for guiding pesticide application, preventing disease spread, and reducing yield losses. Because the model contains only 1.831 M parameters and requires relatively low computational resources, it is, in principle, compatible with mobile phones, drones, and other lightweight edge devices commonly used in agricultural monitoring systems.

In practical farming scenarios, farmers and field technicians often rely on visual assessment, which is prone to subjective bias and inconsistent interpretations. The proposed lightweight model therefore has the potential to provide more objective support for field diagnosis and decision-making. However, the present work evaluates I-GhostNetV3 only in an offline setting; no real on-device or IoT deployment experiments on UAVs, smartphones, or embedded accelerators were conducted. Consequently, any statements about deployment and real-time performance should be regarded as prospective, and systematic hardware-in-the-loop tests and large-scale field trials are left for future work.

### Limitations and Future Work

Although the experimental results are encouraging, several limitations of this study should be acknowledged. First, the main evaluation is conducted on the RLBF rice leaf dataset collected under tropical field conditions in Bangladesh and on a single additional corn dataset (PlantVillage–Corn) from a controlled environment. The limited dataset size, geographic coverage, and domain diversity mean that the reported improvements should be interpreted as preliminary rather than definitive evidence of broad generalization across climates, phenological stages, and sensor types. Future work will include evaluation on independent field datasets from different regions/seasons and acquisition devices.

Second, all experiments rely exclusively on RGB imagery. While RGB cameras are widely available and cost-effective, they cannot capture subtle physiological or spectral changes that multispectral, hyperspectral, or thermal sensors can provide, and very early or spectrally subtle symptoms may remain difficult to detect. Future work will explore multi-modal sensing or lightweight fusion strategies when such sensors are available.

Third, although I-GhostNetV3 is substantially more compact than several comparison models, its computational cost is still higher than that of ultra-compact baselines such as MobileNetV3-Small, which may be a concern for extremely resource-limited devices. Future work will investigate further compression (e.g., pruning/quantization) and accuracy–latency trade-offs for stricter deployment budgets.

Finally, the current work evaluates the model in an offline setting only. No real on-device or IoT deployment experiments on UAVs, smartphones, or edge accelerators were conducted. Consequently, any statements about deployment should be viewed as prospective, and systematic on-device profiling, hardware-in-the-loop tests, and large-scale field trials are left for future work.

## 5. Conclusions

In this paper, we propose I-GhostNetV3, an accuracy-enhanced lightweight CNN built upon a reconfigured GhostNetV3 backbone for rice leaf disease recognition on a field-like benchmark (RLBF). The backbone reconfiguration provides the primary efficiency gain relative to the original GhostNetV3, while two plug-in attention modules (APA and FCCA) are introduced to strengthen lesion-related fine-grained representations under cluttered backgrounds and varying illumination. In particular, APA incurs noticeable computational overhead and is therefore selectively inserted, whereas FCCA serves as a small-overhead replacement of SE to improve spatial–channel interaction.

Experiments on RLBF show that I-GhostNetV3 achieves 90.02% Top-1 accuracy with 1.831 M parameters and 248.694 MFLOPs, showing gains over several widely used lightweight CNN baselines under our experimental setup. Compared with the ultra-light backbone-only configuration, the full model deliberately increases computation to obtain accuracy gains, reflecting an explicit accuracy–efficiency trade-off; meanwhile, it remains substantially more compact than the original GhostNetV3. A Rice-to-Corn transfer experiment on PlantVillage–Corn (a relatively controlled dataset) is further used as a complementary sanity check, suggesting that the proposed modules do not degrade adaptation to a simpler target domain under the current setting. Overall, the results indicate that I-GhostNetV3 is a promising efficient backbone for future embedded/edge deployment; however, the reported latency is measured on an RTX 3080 Ti GPU, and on-device profiling and independent multi-site field validation remain future work.

Future work will expand evaluation to broader multi-site field datasets and conduct on-device experiments. We also plan to integrate semi-supervised/self-supervised learning and domain generalization techniques to further improve robustness under domain shifts and reduce reliance on large-scale annotations.

## Figures and Tables

**Table 1:** Information on the Rice Disease Dataset.

Disease Name	Sample Size	Train+Val	Test	Lesion Characteristics
Bacterial Leaf Blight	164	147	17	Yellowed leaf tip with elongated yellow–brown streaks
Brown Spot	267	241	26	Multiple round brown spots with darker margins
Healthy Rice Leaf	173	155	18	Uniform green leaf without visible lesions
Leaf Blast	305	275	30	Spindle-shaped lesions with gray centers and dark borders
Leaf Scald	189	170	19	Long straw-colored streaks or patches from leaf edge
Narrow Brown Leaf Spot	117	106	11	Very narrow dark-brown streaks along the veins
Rice Hispa	215	194	21	Whitish scraped streaks parallel to the veins
Sheath Blight	271	244	27	Gray-white patches with brown margins on sheaths/leaves

**Table 2:** Structured comparison of APA/FCCA with representative attention blocks (conceptual positioning).

Block	Primary Cue	Extra Guidance	Fusion/Gating	Deployment	Limitation Addressed in GhostNetV3
SE [24]	Channel (GAP+FC)	None	Channel re-scaling	Inside bottleneck	No explicit spatial/edge cue
ECA [21]	Channel (1D conv)	None	Channel re-scaling	Inside bottleneck	Lightweight channel- only recalibration
CA [22]	Coordinate + channel	None	Axis-aware gating	Inside bottleneck	Adds position cues but limited cross-channel dependency modeling
CBAM [25]	Channel + spatial	None	Channel & spatial sequential attention	Inside bottleneck	Spatial attention available but no explicit lesion- boundary guidance
EGA [20]	Spatial/edge	Edge map + early prediction	Spatial guidance	Stage-level enhancement	Boundary/lesion saliency without channel recalibration
APA (Ours)	Spatial + channel	Edge map + early prediction	Complementary channel-wise gating between EGA and ECA branches	Inserted at selected stage outputs	Compensates downsampling-induced boundary loss in cluttered field backgrounds
FCCA (Ours)	Coordinate + channel	None	Per-channel soft fusion between CA-only and (CA + ECA) paths	Replaces SE in bottlenecks	Suppresses background responses with very small overhead relative to SE

**Note:** This table provides a conceptual positioning and is not intended to be exhaustive.

**Table 3:** Experimental setup comparison between the baseline model and I-GhostNetV3.

Parameter	Baseline	I-GhostNetV3	Explanation
Input shape	3×224×224	-	Uniform input resolution
Batch size	32	-	Uniform batch size
Number of training rounds	150	-	Training rounds remain consistent
Optimizer	AdamW	AdamW	Unified optimization algorithm
Initial learning rate	0.0015	0.0015	Consistent learning rate
Weight decay	$2\times 10^{-4}$	$2\times 10^{-4}$	Weights remain consistent
Regularization	Dropout = 0.2	Dropout = 0.2	Regularization remains consistent
Data augmentation	Random rotation, horizontal flip, color disturbance	Same as baseline	Basic enhancements remain consistent
Mixup, CutMix	0.1, 0.3	0.1, 0.3	Identical strong augmentation for fairness
Mixed precision	✓	✓	GPU-side training acceleration (AMP)

**Note:** ✓ indicates that Automatic Mixed Precision (AMP) is enabled during training.

**Table 4:** Performance comparison of I-GhostNetV3 and representative lightweight models on the Rice Leaf Bacterial and Fungal Disease dataset.

Model	Acc (%)	Std (Acc)	Prec (%)	Recall (%)	F1 (%)	FLOPs/M	Params/M	Lat. (ms/img)	Thr. (img/s)
GhostNetV3 (1.3×)	85.53±0.24	0.26	85.61±0.58	84.49±0.48	84.73±0.33	269.000	8.754	1.450	688.22
MobileNetV2-100	86.39±0.26	0.26	85.99±0.13	85.64±0.36	85.73±0.33	299.569	3.415	0.260	3863.28
MobileNetV3-Large	81.86±0.27	0.29	81.16±0.23	80.81±0.33	80.48±0.25	218.358	4.178	0.290	3410.54
MobileNetV3-Small	86.26±0.21	0.23	86.58±0.32	85.21±0.16	85.72±0.35	56.397	2.417	0.240	4103.39
EfficientNet-B0	86.78±0.48	0.52	86.16±0.46	85.61±0.35	85.82±0.44	386.993	5.251	0.380	2627.72
InceptionV3	88.92±0.31	0.33	89.30±0.59	88.18±0.21	88.84±0.52	2859.000	23.768	0.480	244.81
DenseNet-121	88.06±0.13	0.14	87.76±0.33	86.90±0.33	86.12±0.41	2880.000	7.978	0.774	1291.60
MobileNetV4-Conv-S (1×)	79.46±0.52	0.57	78.06±0.17	78.01±0.20	78.01±0.22	184.774	2.468	0.220	4502.55
RepViT-M1.0	86.72±0.43	0.47	86.51±0.44	86.26±0.31	86.18±0.23	1142.000	6.412	0.514	1945.79
I-GhostNetV3 (Ours)	90.02±0.12	0.13	89.71±0.25	88.97±0.24	89.56±0.21	248.694	1.831	1.270	787.76

**Note:** Precision, Recall, and F1-score are macro-averaged across all classes. Latency/throughput are measured on an RTX 3080 Ti GPU (batch size = 1) and are reported as GPU-side proxies, not edge-device results.

**Table 5:** McNemar test results (with continuity correction, df = 1) between I-GhostNetV3 and selected models on the RLBF test set.

Comparison	$n_{10}$	$n_{01}$	$n_{10}\;+\;n_{01}$	$\chi^{2}$	*p*-Value	Sig. (α=0.05)
I-GhostNetV3 vs. GhostNetV3 (1.3×)	29	12	41	6.2439	0.0125	*
I-GhostNetV3 vs. MobileNetV2-100	27	13	40	4.2250	0.0398	*
I-GhostNetV3 vs. InceptionV3	20	15	35	0.4571	0.4990	n.s.

**Note:** *n*_10_ denotes the number of samples misclassified by the baseline model but correctly classified by I-GhostNetV3; *n*_01_ denotes the number of samples correctly classified by the baseline model but misclassified by I-GhostNetV3. “Sig.“ indicates significance (* if *p* < 0.05, n.s. otherwise).

**Table 6:** Ablation study of backbone reconfiguration and attention modules on the Rice Leaf Bacterial and Fungal Disease dataset.

Model	Backbone Reconfiguration	ECA	CA	APA	APA-Lite	FCCA	Params (M)	MFLOPs	Acc (%)
GhostNetV3 (1.3×)	-	-	-	-	-	-	8.754	269.000	85.53±0.24
GhostNetV3 + Backbone	✓	-	-	-	-	-	0.383	59.555	86.26±0.12
GhostNetV3 + Backbone + ECA	✓	✓	-	-	-	-	0.386	59.939	86.37±0.21
GhostNetV3 + Backbone + CA	✓	-	✓	-	-	-	0.401	60.716	86.37±0.11
GhostNetV3 + Backbone + APA	✓	-	-	✓	-	-	0.548	85.970	87.70±0.12
GhostNetV3 + Backbone + APA-lite	✓	-	-	-	✓	-	0.498	65.539	87.45±0.22
GhostNetV3 + Backbone + FCCA	✓	-	-	-	-	✓	0.342	59.842	87.82±0.11
I-GhostNetV3 (Ours)	✓	-	-	${✓}^{†}$	-	✓	1.831	248.694	90.02±0.12

**Note:** Acc is reported as mean ± 95% confidence interval over 7 independent runs. The 95% confidence interval is computed as $\bar{x}\pm t_{0.975,\;6}\cdot \frac{s}{\sqrt{7}}$, where $\bar{x}$ and *s* are the sample mean and standard deviation, respectively, and $t_{0.975,\;6}$ is the *t*-value with 6 degrees of freedom. ✓ indicates replacing the original SE blocks with the corresponding module (SE→ECA/CA/APA/APA-lite/FCCA). ${✓}^{†}$ indicates additionally inserting APA on top of the backbone where SE has been replaced by FCCA (i.e., FCCA replacement + APA insertion in the final model).

**Table 7:** Percentage of computation attributed to the edge-guided branch in APA.

Setting	Total MFLOPs	Δ MFLOPs	Edge/APA (%)	Edge/Total (%)
Backbone-only (with SE)	59.555	-	-	-
Backbone + APA	85.970	26.415	78.4	24.1

**Note:** The *edge module* refers to the EGA-driven edge-guided spatial branch inside APA (input: 224×224, batch size = 1, eval mode). FLOPs are computed using the PyTorch(v12.6) thop library; minor discrepancies may arise from non-convolutional operations. Edge/APA (%) is computed as the fraction of the *measured (profiled)* MFLOPs within APA attributed to the edge-guided branch, while Edge/Total (%) is computed with respect to the total MFLOPs of the Backbone + APA setting (where APA replaces SE).

**Table 8:** Cross-Domain Transfer Performance of Different Lightweight Models (Rice→Corn).

Model	Accuracy (%)	Precision (%)	Recall (%)	F1-Score (%)	Δ Accuracy
GhostNetV3	97.82±0.21	97.32±0.26	97.27±0.16	97.18±0.35	↑
MobileNetV4-Conv-S (1×)	97.05±0.22	96.82±0.34	95.72±0.21	96.12±0.14	↑
RepViT-M1.0	96.22±0.11	95.22±0.25	94.82±0.32	94.95±0.13	↑
I-GhostNetV3 (Ours)	98.22±0.16	97.92±0.31	97.86±0.26	97.89±0.29	↑

**Note:** ↑ indicates an increase in accuracy relative to the Rice dataset.

**Table 9:** Per-class performance derived from the confusion matrix on the RLBF test set (single run, N = 252).

Class	Precision	Recall	F1-Score	Support
0	0.9524	0.7407	0.8333	27
1	0.9211	0.8750	0.8974	40
2	0.8400	0.9130	0.8750	23
3	0.9130	0.9333	0.9231	45
4	0.8065	0.8929	0.8475	28
5	0.8333	0.8824	0.8571	17
6	0.9375	0.9375	0.9375	32
7	0.8780	0.9000	0.8889	40
**Macro Avg.**	**0.8852**	**0.8844**	**0.8825**	–
**Weighted Avg.**	**0.8922**	**0.8889**	**0.8886**	–
**Micro Avg.**	**0.8889**	**0.8889**	**0.8889**	252

**Note:** Bold values in the table represent the summary statistics for different averaging methods (Macro Avg., Weighted Avg., Micro Avg.), with bold highlighting used for emphasis. The Micro Avg. corresponds to accuracy in the case of single-label multi-class classification.

## Data Availability

The data used in this study are publicly available. The Rice Leaf Bacterial and Fungal Disease Dataset can be accessed at https://data.mendeley.com/datasets/hx6f852hw4/2 (accessed on 20 July 2025), and the PlantVillage-Corn Dataset is available at https://github.com/gabrieldgf4/PlantVillage-Dataset (accessed on 27 August 2025). These datasets are used for plant disease classification and model evaluation in cross-domain transfer learning.

## References

[1] Peng S., Huang J., Sheehy J.E., Laza R.C., Visperas R.M., Zhong X., Centeno G.S., Khush G.S., Cassman K.G. (2004). Rice yields decline with higher night temperature from global warming. Proc. Natl. Acad. Sci. USA.

[2] Fadah I., Lutfy C., Amruhu A. (2024). Analysis of rice trade and food security in southeast asian countries. KnE Soc. Sci..

[3] Joshi A.A., Jadhav B.D. (2016). Monitoring and controlling rice diseases using image processing techniques. Proceedings of the 2016 International Conference on Computing, Analytics and Security Trends (CAST), Pune, India, 19–21 December 2016.

[4] Barbedo J.G.A. (2013). Digital image processing techniques for detecting, quantifying and classifying plant diseases. SpringerPlus.

[5] Mahlein A.-K. (2016). Plant disease detection by imaging sensors–parallels and specific demands for precision agriculture and plant phenotyping. Plant Dis..

[6] Cao X., Liu Y., Yu R., Han D., Su B. (2021). A comparison of uav rgb and multispectral imaging in phenotyping for stay green of wheat population. Remote Sens..

[7] Nguyen C., Sagan V., Maimaitiyiming M., Maimaitijiang M., Bhadra S., Kwasniewski M.T. (2021). Early detection of plant viral disease using hyperspectral imaging and deep learning. Sensors.

[8] Upadhyay A., Chandel N.S., Singh K.P., Chakraborty S.K., Nandede B.M., Kumar M., Subeesh A., Upendar K., Salem A., Elbeltagi A. (2025). Deep learning and computer vision in plant disease detection: A comprehensive review of techniques, models, and trends in precision agriculture. Artif. Intell. Rev..

[9] Kahar M.A., Mutalib S., Abdul-Rahman S. (2015). Early detection and classification of paddy diseases with neural networks and fuzzy logic. Proceedings of the 17th International Conference on Mathematical and Computational Methods in Science and Engineering (MACMESE), Istanbul, Turkey, 2–7 August 2015.

[10] Singh A.K., Rubiya A., Raja B.S. (2015). Classification of rice disease using digital image processing and SVM classifier. Int. J. Electr. Electron. Eng..

[11] Rahman C.R., Arko P.S., Ali M.E., Khan M.A.I., Apon S.H., Nowrin F., Wasif A. (2020). Identification and recognition of rice diseases and pests using convolutional neural networks. Biosyst. Eng..

[12] Patel B., Sharaff A. (2023). Automatic rice plant disease diagnosis using gated recurrent network. Multimed. Tools Appl..

[13] Haque M.E., Rahman A., Junaeid I., Hoque S.U., Paul M. (2022). Rice leaf disease classification and detection using YOLOv5. arXiv.

[14] Sharma S., Vardhan M. (2025). Aelgnet: Attention-based enhanced local and global features network for medicinal leaf and plant classification. Comput. Biol. Med..

[15] Cheng C., Wang Y., Song Q., Jian C., Zhang Y., Wei Z. (2025). Lightweight ECA-ResNeXt model for smart agriculture and application in rice disease identification. Agric. Eng..

[16] Elakya R., Manoranjitham T. (2022). Classification of diseases in paddy by using deep transfer learning MobileNetV2 model. Proceedings of the 2022 1st International Conference on Computational Science and Technology (ICCST), Chennai, India, 9–10 November 2022.

[17] Asvitha S., Dhivya T., Dhivyasree H., Bhavadharini R.M. (2022). Paddy pro: A MobileNetV3-based app to identify paddy leaf diseases. Proceedings of the International Conference on Computing, Communications, and Cyber-Security, Ghaziabad, India, 21–22 October 2022.

[18] Hasan M., Khatun S., Raihan M.A., Uddin A.H. (2023). Rice leaf bacterial and fungal disease dataset. Mendeley Data.

[19] Mohanty S.P., Hughes D.P., Salathé M. (2016). Using deep learning for image-based plant disease detection. Front. Plant Sci..

[20] Bui N.-T., Hoang D.-H., Nguyen Q.-T., Tran M.-T., Le N. (2024). Meganet: Multi-scale edge-guided attention network for weak boundary polyp segmentation. Proceedings of the IEEE/CVF Winter Conference on Applications of Computer Vision (WACV), Waikoloa, HI, USA, 3–8 January 2024.

[21] Wang Q., Wu B., Zhu P., Li P., Zuo W., Hu Q. (2020). ECA-Net: Efficient channel attention for deep convolutional neural networks. Proceedings of the IEEE/CVF Conference on Computer Vision and Pattern Recognition (CVPR), Seattle, WA, USA, 14–19 June 2020.

[22] Hou Q., Zhou D., Feng J. (2020). Coordinate attention for efficient mobile network design. Proceedings of the IEEE/CVF Conference on Computer Vision and Pattern Recognition, Nashville, TN, USA, 19–25 June 2021.

[23] Liu Z., Hao Z., Han K., Tang Y., Wang Y. (2024). GhostNetV3: Exploring the training strategies for compact models. arXiv.

[24] Hu J., Shen L., Sun G. (2018). Squeeze-and-excitation networks. Proceedings of the IEEE Conference on Computer Vision and Pattern Recognition (CVPR), Salt Lake City, UT, USA, 18–22 June 2018.

[25] Woo S., Park J., Lee J.-Y., Kweon I.S. (2018). CBAM: Convolutional block attention module. Proceedings of the European Conference on Computer Vision (ECCV), Munich, Germany, 8–14 September 2018.

[26] Sandler M., Howard A., Zhu M., Zhmoginov A., Chen L.-C. (2018). MobileNetV2: Inverted residuals and linear bottlenecks. Proceedings of the IEEE Conference on Computer Vision and Pattern Recognition (CVPR), Salt Lake City, UT, USA, 18–22 June 2018.

[27] Howard A., Sandler M., Chu G., Chen L.-C., Chen B., Tan M., Wang W., Zhu Y., Pang R., Vasudevan V. (2019). Searching for MobileNetV3. Proceedings of the IEEE/CVF International Conference on Computer Vision (ICCV), Seoul, Republic of Korea, 27 October–2 November 2019.

[28] Tan M., Le Q.V. Efficientnet: Rethinking model scaling for convolutional neural networks. Proceedings of the 36th International Conference on Machine Learning (ICML), Long Beach, CA, USA, 9–15 June 2019.

[29] Szegedy C., Vanhoucke V., Ioffe S., Shlens J., Wojna Z. (2016). Rethinking the inception architecture for computer vision. Proceedings of the IEEE Conference on Computer Vision and Pattern Recognition (CVPR), Las Vegas, NV, USA, 27–30 June 2016.

[30] Huang G., Liu Z., van der Maaten L., Weinberger K.Q. (2017). Densely connected convolutional networks. Proceedings of the IEEE Conference on Computer Vision and Pattern Recognition (CVPR), Honolulu, HI, USA, 21–26 July 2017.

[31] Qin D., Leichner C., Delakis M., Fornoni M., Luo S., Yang F., Wang W., Banbury C., Ye C., Akin B. (2025). MobileNetV4: Universal models for the mobile ecosystem. Proceedings of the Computer Vision–ECCV 2024 Lecture Notes in Computer Science, Milan, Italy, 29 September–4 October 2024.

[32] Wang A., Chen H., Lin Z., Han J., Ding G. (2024). Repvit: Revisiting mobile CNN from ViT perspective. Proceedings of the IEEE/CVF Conference on Computer Vision and Pattern Recognition (CVPR), Seattle, WA, USA, 16–22 June 2024.

[33] Chattopadhay A., Sarkar A., Howlader P., Balasubramanian V.N. (2018). Grad-CAM++: Generalized gradient-based visual explanations for deep convolutional networks. Proceedings of the 2018 IEEE Winter Conference on Applications of Computer Vision (WACV), Lake Tahoe, NV, USA, 12–15 March 2018.

[34] Islam T., Sah M., Baral S., Choudhury R.R. (2018). A faster technique on rice disease detection using image processing of affected area in agro-field. Proceedings of the 2018 Second International Conference on Inventive Communication and Computational Technologies (ICICCT), Coimbatore, India, 20–21 April 2018.

[35] Kiratiratanapruk K., Temniranrat P., Kitvimonrat A., Sinthupinyo W., Patarapuwadol S. (2020). Using deep learning techniques to detect rice diseases from images of rice fields. Proceedings of the International Conference on Industrial, Engineering and Other Applications of Applied Intelligent Systems, Kitakyushu, Japan, 21–24 July 2020.

[36] Li P., Zhou J., Sun H., Zeng J. (2025). Rdrm-YOLO: A high-accuracy and lightweight rice disease detection model for complex field environments based on improved YOLOv5. Agriculture.

[37] Wang Y., Wang H., Peng Z. (2021). Rice diseases detection and classification using attention based neural network and bayesian optimization. Expert Syst. Appl..

[38] Ni H., Shi Z., Karungaru S., Lv S., Li X., Wang X., Zhang J. (2023). Classification of typical pests and diseases of rice based on the ECA attention mechanism. Agriculture.

[39] Jiang M., Feng C., Fang X., Huang Q., Zhang C., Shi X. (2023). Rice disease identification method based on attention mechanism and deep dense network. Electronics.

